# Did Clinical Trials in Which Erythropoietin Failed to Reduce Acute Myocardial Infarct Size Miss a Narrow Therapeutic Window?

**DOI:** 10.1371/journal.pone.0034819

**Published:** 2012-04-18

**Authors:** Mark I. Talan, Ismayil Ahmet, Edward G. Lakatta

**Affiliations:** Laboratory of Cardiovascular Sciences, Intramural Research Program, National Institute on Aging, National Institutes of Health, Baltimore, Maryland, United States of America; Cliniche Humanitas Gavazzeni, Italy

## Abstract

**Background:**

To test a hypothesis that in negative clinical trials of erythropoietin in patients with acute myocardial infarction (MI) the erythropoietin (rhEPO) could be administered outside narrow therapeutic window. Despite overwhelming evidence of cardioprotective properties of rhEPO in animal studies, the outcomes of recently concluded phase II clinical trials have failed to demonstrate the efficacy of rhEPO in patients with acute MI. However, the time between symptoms onset and rhEPO administration in negative clinical trials was much longer that in successful animal experiments.

**Methodology/Principal Findings:**

MI was induced in rats either by a permanent ligation of a descending coronary artery or by a 2-hr occlusion followed by a reperfusion. rhEPO, 3000 IU/kg, was administered intraperitoneally at the time of reperfusion, 4 hrs after beginning of reperfusion, or 6 hrs after permanent occlusion. MI size was measured histologically 24 hrs after coronary occlusion. The area of myocardium at risk was similar among groups. The MI size in untreated rats averaged ∼42% of area at risk, or ∼24% of left ventricle, and was reduced by more than 50% (p<0.001) in rats treated with rhEPO at the time of reperfusion. The MI size was not affected by treatment administered 4 hrs after reperfusion or 6 hrs after permanent coronary occlusion. Therefore, our study in a rat experimental model of MI demonstrates that rhEPO administered within 2 hrs of a coronary occlusion effectively reduces MI size, but when rhEPO was administered following a delay similar to that encountered in clinical trials, it had no effect on MI size.

**Conclusions/Significance:**

The clinical trials that failed to demonstrate rhEPO efficacy in patients with MI may have missed a narrow therapeutic window defined in animal experiments.

## Introduction

During the last decade powerful cardioprotective properties of exogenous recombinant human erythropoietin (rhEPO) have been demonstrated in numerous experimental studies of two different models of myocardial infarction (MI), a permanent coronary ligation and ischemia/reperfusion model, in several species [1]–[6]. Despite positive outcomes observed in vast majority of animal experiments, the results of several, phase II, clinical trials in humans concluded recently (Table 1) were far less than encouraging [7]–[19]. In fact, in an editorial in JAMA [20] written in conjunction with publication of results of the recent clinical trial with a negative outcome, “Reduction of Infarct Expansion and Ventricular Remodeling With Erythropoietin After Large Myocardial Infarction” [18], recommended that no future clinical trials of EPO should be undertaken. Moreover, it implied that frequently occurring discrepancies between successful animal experiments and negative outcomes of clinical trials in humans, in context of reduction of MI size, make it impractical to conduct clinical trials based on animal studies; but rather promising new therapies should be screened in small number of patients.

**Table 1:** Clinical trials. Erythropoietin in treatment of acute MI.

Name/year/reference	Country	# of pt	EPO, type and dose	Average time to drug		Primary endpoint		Secondary endpoint	
				From onset	From PCI	Measurements	Outcome	Measurements	Outcome
Lipcic 2006 (7)	Netherland	20	Darbepoetin alfa 60,000 IU	NA	0 min	LVEF at 4 m	NS	NA	NA
Liem, 2009 (8)	Netherland	51	Epoetin alfa 40,000 IU	8 h from elevated troponin	NA	MI size (enzymes)	NS	NA	NA
Binbrec, 2009 (9, 19)	Dubai	236	Epoetin beta 30,000 IU	6 h	At the time of thrombolysis	MI size (enzymes)	NS	Echo at discharge	NS
EPO/AMI-1, 2010 (11, 12)	Japan	36	Epoetin beta 12,000 IU	Within 48 h	Within 24 h	LVEF 4 d and 6 m	NS	NA	NA
HEBE III 2010 (13, 14)	Netherland, Germany	529	Epoetin alfa 60,000 IU	12–24 h	Within 3 h	LVEF at 6 w	NS	MI size (enzymes)	NS
REVIVAL-3, 2010 (15)	Germany	138	Epoetin beta 33,300 IU	Within 24 h	0 min (Add. doses at 24 and 48 h)	LVEF at 6 m	NS	ΔLVEF and MI	NS
EPOC-AMI, 2010 (16)	Japan	35	Epoetin beta 6,000 IU	Within 24 h	0 min (Add. doses at 24 and 48 h)	MI size 4 d and 6 m	NS	NA	NA
REVEAL, 2011 (17, 18)	USA	131	Epoetin alfa 60,000 IU	Within 8 h	Within 4 h	MI size 2 to 6 d and 12 w	NS	LV remodeling	NS
Ferrario et al, 2011 (19)	Italy	30	Epo 33,000 IU	Within 6 h	0 min (Add. doses at 24 and 48 h)	MI size (enzymes)LVEF at admission and at 6 m	MI **↓** p<0.02 EF **↑** p<0.05	Progen. cells at 72 hr	**↑** p<.002

But what if a main reason for failure to translate animal experiment findings into a clinical application was not due to fundamental differences between human and animal species in response to EPO but rather due to differences in designs between preclinical (animal) and clinical (human) trials. An analysis of literature on animal experiments related to myocardial protection from ischemic damage by erythropoietin [1]–[6], as well as dissection of details of design of several completed clinical trials [7]–[19], clearly indicates that most of these clinical trials have a common trend – the time interval between presumed occlusion of a coronary artery (onset of symptoms) and drug administration was much greater in human clinical trials with a negative outcome than described in any animal experiment with a positive outcome. Thus, in the case of erythropoietin in patients with an acute MI, a reason why rhEPO was ineffective to reduce an MI size may have been the delay in drug administration. We investigated the role of such a delay in therapy in two rat models of MI induced by a permanent or temporary ligation of a coronary artery, in which we followed the design of the last clinical trial, REVEAL [17], [18]. Specifically, we compared the MI size among three different situations: when the bolus injections of rhEPO were administered systemically into rats either 2 hours after coronary ligation at the time of reperfusion, or 4 hours after reperfusion that followed 2 hours of coronary occlusion, or 6 hours after permanent coronary occlusion without reperfusion. A significant reduction of MI size was observed when rhEPO was administered 2 hrs after coronary ligation at the time of reperfusion; Delaying administration of EPO treatment to 4 hours after reperfusion or 6 hours after permanent coronary occlusion abolished its effect to reduce an MI size.

## Methods

**Figure 1:**
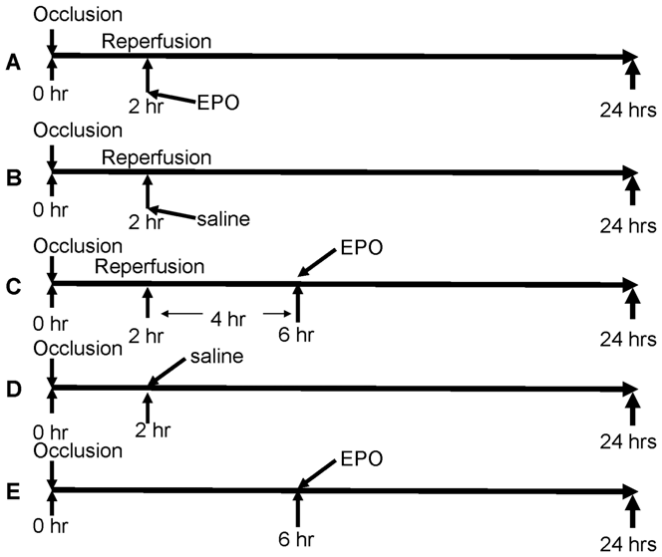
Diagram of experimental design. In A, B, and C left descending coronary artery is occluded for 2 hrs and reperfused for 22 hrs. In D and E left descending coronary artery is occluded for 24 hrs. rhEPO administered 2 hrs after a temporary coronary occlusion at the time of reperfusion (A), or 6 hrs after a temporary coronary occlusion, i.e., 4 hrs after beginning of reperfusion (C), or 6 hrs after a permanent coronary occlusion (E). Physiological saline administered 2 hrs after a temporary occlusion at the time or reperfusion (B) or 2 hrs after a permanent occlusion (D).

2-mo old male Wistar rats, obtained from Charles River Laboratories (Wilmington, MA), were studied in conformance with the NIH Guide for the Care and Use of Laboratory Animals, Manual 3040–2 (1996), with NIA Animal Care and Use Committee approval. Rats were divided into 5 experimental groups, 10 animals in each. All rats were subjected to open chest surgery under Isoflurane anesthesia (2% in oxygen) - coronary artery ligation [21] - and were euthanized 24 hrs after beginning of coronary occlusion for histological evaluation of infarct size. The experimental design is illustrated in the Figure 1. In 2 groups (D, E) the ligature occluding coronary artery remained in place for 24 hrs, in 3 groups (A, B, C) the ligature was released after 2 hrs of occlusion and the myocardium was reperfused. Rats from one of the permanent occlusion groups (D) and one of the ischemia/reperfusion (I/R) groups (B) remained untreated (injected intraperitoneally with 0.5 ml of saline 2 hrs after coronary occlusion). Rats from the second I/R group (A) had rhEPO (3000 IU/kg) administered at the time of reperfusion, i.e., 2 hrs after occlusion. Rats from the third I/R group (C) had rhEPO injected 4 hrs after reperfusion, i.e., 6 hrs after occlusion. In the second permanent occlusion group rhEPO was also injected 6 hrs after coronary occlusion. Myocardial area at risk (AAR) and MI size were measured as previously described [22]. Briefly, 3 mL of 5% Evans blue dye (Sigma) was rapidly injected into the aorta via a 16-gauge tube to distinguish the perfused area (blue staining) from the unperfused area (no staining). The atria and great vessels were dissected away from the heart, and the heart was cut transversely into 4 slices from base to apex. Heart samples were incubated at 37°C in 4% triphenyltetrazolium chloride (TTC, Sigma) for 30 minutes to distinguish the infarct area (unstained) from the AAR (brick red staining) in the unperfused area. All images were analyzed with NIH Image software. MI size was expressed as a percentage of the unperfused area or LV. Differences among groups were statistically evaluated using one-way analyses of variance with post-hoc Bonferroni comparison.

## Results

**Figure 2:**
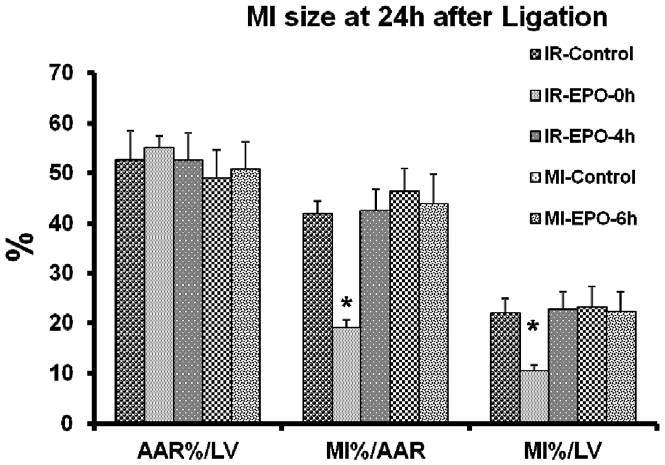
Size of myocardial infarction. Area of myocardium at risk (AAR) and size of myocardial infarction (MI) 24 hrs after permanent occlusion of the left descending coronary artery or after 2 hrs of occlusion followed by 22 hrs of reperfusion. rhEPO is administered at the time of reperfusion (IR-EPO-0 h) or 4 hrs after beginning of reperfusion (IR-EPO-4 h) or 6 hrs after permanent coronary occlusion (MI-EPO-6 h). Data presented as means ± SEM. AAR is presented as % of left ventricle (LV). MI is presented as % of AAR or % of LV. *- p<0.05 vs all other group (Bonferroni post hoc comparison).

MI size 24 hrs after coronary occlusion is illustrated in Figure 2. AAR was similar in all 5 groups. MI size, expressed either as a percent of AAR, or as a percent of left ventricle was similar between untreated I/R and permanent occlusion groups and averaged 42±2% of the AAR or ∼24% of LV. MI size in the group treated at the time of reperfusion was reduced by more than 50% (19±2% of the AAR or 9% of LV, p<0.0001). However, MI size in groups in which EPO treatment was delayed by 4 hrs after reperfusion or by 6 hrs after permanent coronary occlusion did not differ from MI size of untreated animals.

## Discussion

In the recently completed clinical trial, REVEAL [17], [18] patients received 60,000 IU of rhEPO (approximately 750 IU/kg) within 4 hrs of percutaneous intervention (PCI) (actually within 8 hrs of symptom onset) and treatment was not effective in reduction of MI size, or in attenuation of cardiac remodeling. In our experiment in a rat model of MI, treatment was very effective, when rhEPO was injected at the time of reperfusion following 2 hrs after coronary occlusion. However, the MI size in rats was completely unaffected by treatment if it were delayed by 6 hrs post occlusion, regardless whether reperfusion was performed or the coronary artery remained occluded. Thus in our animal experiment, designed to imitate the design of the REVEAL clinical trial, application of rhEPO failed to reduce the MI size. Most of other clinical trials (see Table 1) have the same design problem as REVEAL, i.e., delayed treatment. Even in trials when EPO was injected at the time of PCI, the time from the symptom onset was sometimes as long as 24 hrs. In the only trial with an encouraging outcome [19], the EPO was injected **at the time of PCI** and the PCI was done within 6 hrs of the beginning of symptoms.

Among numerous experimental studies of cardioprotection by rhEPO, very few were specifically designed to establish a therapeutic window. But in one study in the rat model of MI induced by a permanent coronary occlusion Moon et al [23] had shown that a high dose of rhEPO (3000 IU/kg) can be effective in reduction of MI size, if injected not later than 12 hrs after coronary occlusion. However, a small dose (150 IU/kg), which is equivalent to 10000–15000 IU for a human of average body weight, was effective **only if administered at the time of occlusion**. Similar results were reported by Hirata et al [24] in experiments with a permanent ligation of a coronary artery in dogs – MI was reduced if treated **at the time of ligation**, but not 6 hrs later. It is necessary to note here that in both these studies the permanent occlusion model was used. In most of experiments reporting beneficial effect of EPO on MI size in animal models of ischemia/reperfusion injury treatment was administered **at the time of reperfusion** [3], [4], i.e., 30–60 min after coronary occlusion.

Positive outcome of therapeutic interventions in experiments on animal models of disease cannot assure that similar interventions will be successful in patients. In contrast, there is no doubt that an intervention that failed to affect improvement in the animal model will be unsuccessful in patients. Therefore, while our findings cannot assure that species differences in response to therapy will not affect the outcome of human clinical trials, they strongly suggest that a reason for failure in rhEPO clinical trials in patients with acute MI might have been a difference in their design, i.e., the timing of drug administration substantially deviated from that of preclinical animal experiments. The discrepancy in design between preclinical and clinical trials was certainly influenced by regulatory and ethical issues justifiably associated with conducting a clinical trial involving a patient in an emergency clinical setting resulting in difficulties to obtain informed consent early enough. Another reason for this discrepancy may be the lack of creative interaction between animal researchers and clinicians planning clinical trial: in an ideal world preclinical animal trials would be designed to imitate, as close as possible, a clinical situation. Conversely, clinicians, aiming to initiate a clinical trial, need to dialog with animal researchers about specific, clinically relevant, details of their experiments and perhaps request that additional experiments in an animal model be conducted to resolve questionable issues prior to finalizing a trial design in humans. As a bare minimum, pharmacodynamics, pharmacokinetics, dose response, and therapeutic window (where applicable) must be established in animal experiments, and this information applied to the design of a clinical trial.

In summary, a prerequisite condition for a proper clinical trial of therapeutic efficacy of EPO in acute MI is a short time between symptom onset and EPO administration. Currently in the USA, the average symptom onset-to-door time among patients with ST-segment elevation infarct is 1.6 hrs and the average door-to-balloon time is 87 minutes [25]. Thus, a total time from the symptom onset to a primary PCI is around 3 hrs, which, according to animal experiment data, approaches or even exceeds the limits of EPO therapeutic window. Therefore, randomization and treatment should take place as soon as possible after ST-elevation is observed and MI is confirmed. If rhEPO were to be administered before a patient receives PCI, the likelihood of accessing the therapeutic time window for EPO would be enhanced.
